# Design of Porous Metal Block Augmentation to Treat Tibial Bone Defects in Total Knee Arthroplasty Based on Topology Optimization

**DOI:** 10.3389/fbioe.2021.765438

**Published:** 2021-11-08

**Authors:** Yang Liu, Bingpeng Chen, Chenyu Wang, Hao Chen, Aobo Zhang, Weihuang Yin, Naichao Wu, Qing Han, Jincheng Wang

**Affiliations:** ^1^ Department of Orthopedics, The Second Hospital of Jilin University, Changchun, China; ^2^ Department of Plastic and Reconstructive Surgery, First Bethune Hospital of Jilin University, Changchun, China; ^3^ Department of Oral and Maxillofacial Surgery, Hospital of Stomatology, Jilin University, Changchun, China

**Keywords:** finite element analysis, total knee arthroplasty, topology optimization, metal block augmentation, bone defect

## Abstract

Metal block augmentation, which is used for the treatment of tibial bone defects in total knee arthroplasty, with high stiffness will cause significant alteration in stress distribution, and its solid structure is not suitable for osseointegration. This study aimed to design a porous block to reduce weight, promote bone ingrowth, and improve its biomechanical performance. The metal block augmentation technique was applied to finite element models of tibial bone defects. Minimum compliance topology optimization subject to volume fraction combined with the porous architecture was adopted to redesign the block. Biomechanical changes compared with the original block were analyzed by finite element analysis. The stress distribution of the block and proximal tibia was recorded. The strain energy density of the proximal tibia was obtained. The newly designed block realized 40% weight reduction. The maximum stress in the optimized block decreased by 11.6% when compared with the solid one. The maximum stress of the proximal tibia in the optimized group increased by 18.6%. The stress of the anterior, medial, and posterior parts of the proximal medial tibia in the optimized group was significantly greater than that in the original group (all *p* < 0.05). The optimized block could effectively improve the biomechanical performance between the block and the bone. The presented method might provide a reference for the design of customized three-dimensional printed prostheses.

## Introduction

Osteoarthritis is the most common joint disease worldwide (Glyn-Jones et al., 2015). Bone defects represent a common condition in patients with severe osteoarthritis, among which peripheral tibial bone defects frequently occur (Tsukada et al., 2013). Total knee arthroplasty (TKA) is one of the most effective surgical operations for pain relief and function recovery in patients with severe osteoarthritis (Choi and Ra, 2016). When severe uncontained tibial bone defects are encountered, it could be difficult to restore the anatomical structure and function with normal tibial components (Lee and Choi, 2011). The metal block augmentation technique is one of the major options to treat uncontained bone defects because of its extensive modularity, large availability, and convenience (Panni et al., 2013). However, the current metal block augmentation technique has its limitations.

The significant alteration in stress distribution is one of the most significant shortcomings. The current metal block augmentation is mostly composed of titanium alloy, which is much stiffer than bone (Peto et al., 2019). The stiffness mismatch between the bone and block might cause significant alteration in stress distribution (Rahimizadeh et al., 2018). The most severe problem caused by this alteration is stress shielding, wherein the implant will cause a large amount of load to be applied over a smaller area (Chuah et al., 2010). As a result, the metal block augmentation causes the surrounding areas of the bone to be relatively unloaded (He et al., 2018). According to Wolff’s law, bone develops a structure that is well-suited to resist any force acting on it (Bugbee et al., 1996). Significant peri-implant bone resorption can occur as a result of stress shielding (Iolascon et al., 2010). Thus, the stiffness mismatch will cause stress shielding, resulting in bone resorption and implant loosening (Wegner et al., 2019). A structural design technique that can make the stress between the implant and bone better distributed needs to be adopted to reduce stress shielding.

Topology optimization (TO) is one such structural design technique which provides the optimal shape of the structure from a prescribed domain subjected to certain design considerations such as loading and boundary conditions (Park et al., 2019). Through the TO technique, the stress between the implant and the bone can be better distributed. Guo et al. redesigned an interspinous device through TO and reported that the load transfer was enhanced (Guo and Yin, 2019). Tamimi designed a fracture fixation device based on TO which decreased stress shielding (Al-Tamimi et al., 2017). On the other hand, the solid structure of the current metal block augmentation is not suitable for osseointegration (Baek and Choi, 2011; Chung et al., 2016). Based on the TO design, the graded lattice structure can further reduce the elasticity modulus of the prosthesis and provide a microenvironment for bone ingrowth while maintaining prosthesis stability (Zhang et al., 2020). Thus, the TO technique was incorporated with porous architecture in this study. Porous architecture has been wildly used in orthopedics as it could effectively reduce stiffness (Ryan et al., 2006; Innocenti et al., 2018; Peto et al., 2019). Wang et al. designed a hip implant with three-dimensional (3D) porous architecture to reduce stress shielding and prevent implant micromotion (Wang et al., 2018). The trabecular metal cups obtained from Zimmer Biomet are made of porous tantalum and have been shown to provide good initial stability and bone ingrowth qualities (Laaksonen et al., 2018). A grid-graded structure could provide not only different stiffnesses but also the space for bone ingrowth. By incorporating TO and a porous architecture, a grid-graded metal block augmentation could be designed to reduce weight, promote bone ingrowth, and improve its biomechanical performance. To our knowledge, no relevant study has yet designed a porous metal block augmentation based on the TO technique.

Therefore, in this study, we aimed to redesign a porous metal block augmentation to reduce weight, promote bone ingrowth, and improve its biomechanical performance. Biomechanical changes compared with the original block were analyzed by finite element analysis (FEA).

## Materials and Methods

### Tibia Model Geometry

The geometry of the tibia model was based on a computed tomography (CT) scan of the lower limbs of a 66-year-old female volunteer (weight: 65 kg) who suffered from right knee osteoarthritis. The CT data were imported to Mimics v21.0 software (Materialise, Leuven, Belgium), and the right tibia was reconstructed into a 3D model. This study was approved by the Ethics Committee of the Second Hospital of Jilin University, and informed consent was obtained from the volunteer.

**FIGURE 1:**
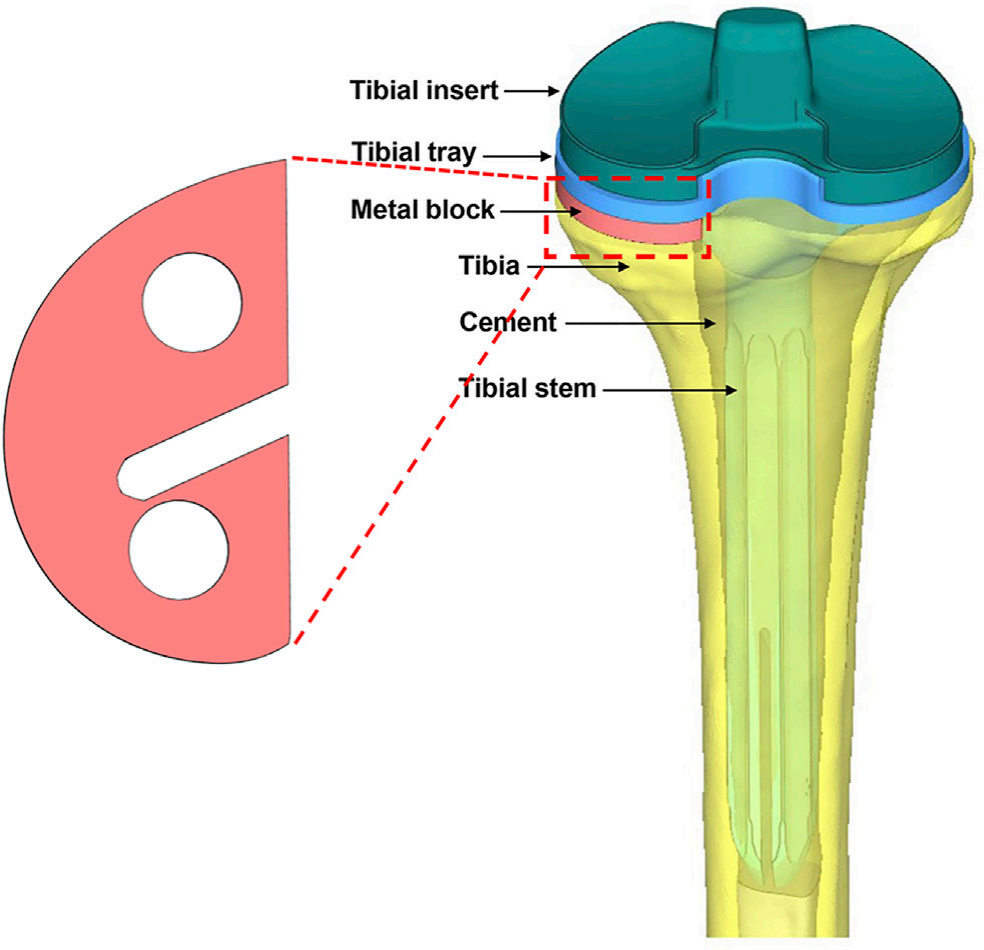
Illustration of the metal block and the entire assembled model from the posterior view. The model consists of a tibial insert, tibial tray, tibial stem, metal block augmentation, bone cement, and bone.

The implants—including the tibial tray (length of the fixation stem: 25.4 mm), extension stem (length: 150 mm), insert (thickness: 7 mm), and metal block augmentation (thickness: 5 mm)—were from the A3 series (AK Medical, Beijing, China). The implants were scanned by a 3D scanner, and the data were processed using Geomagic Studio v2013 (3D Systems, Rock Hill, SC, United States). The tibia model was resected according to the traditional surgical procedure (Bathis et al., 2004; Kang et al., 2019). Briefly, the tibia was first resected 8 mm below its medial articular surface, perpendicular to its mechanical axis. Then, the model was resected to achieve a 6° posterior slope of the baseplate of the tibial component for TKA. Additional resections were performed to generate the uncontained medial tibial bone defect measuring 5 mm in depth. Then, the bone cement layer was used to increase fixation between the implants and bone, as well as between the metal block and the tibial tray. Finally, one set of assembled finite element models was created (Figure 1).

**FIGURE 2:**
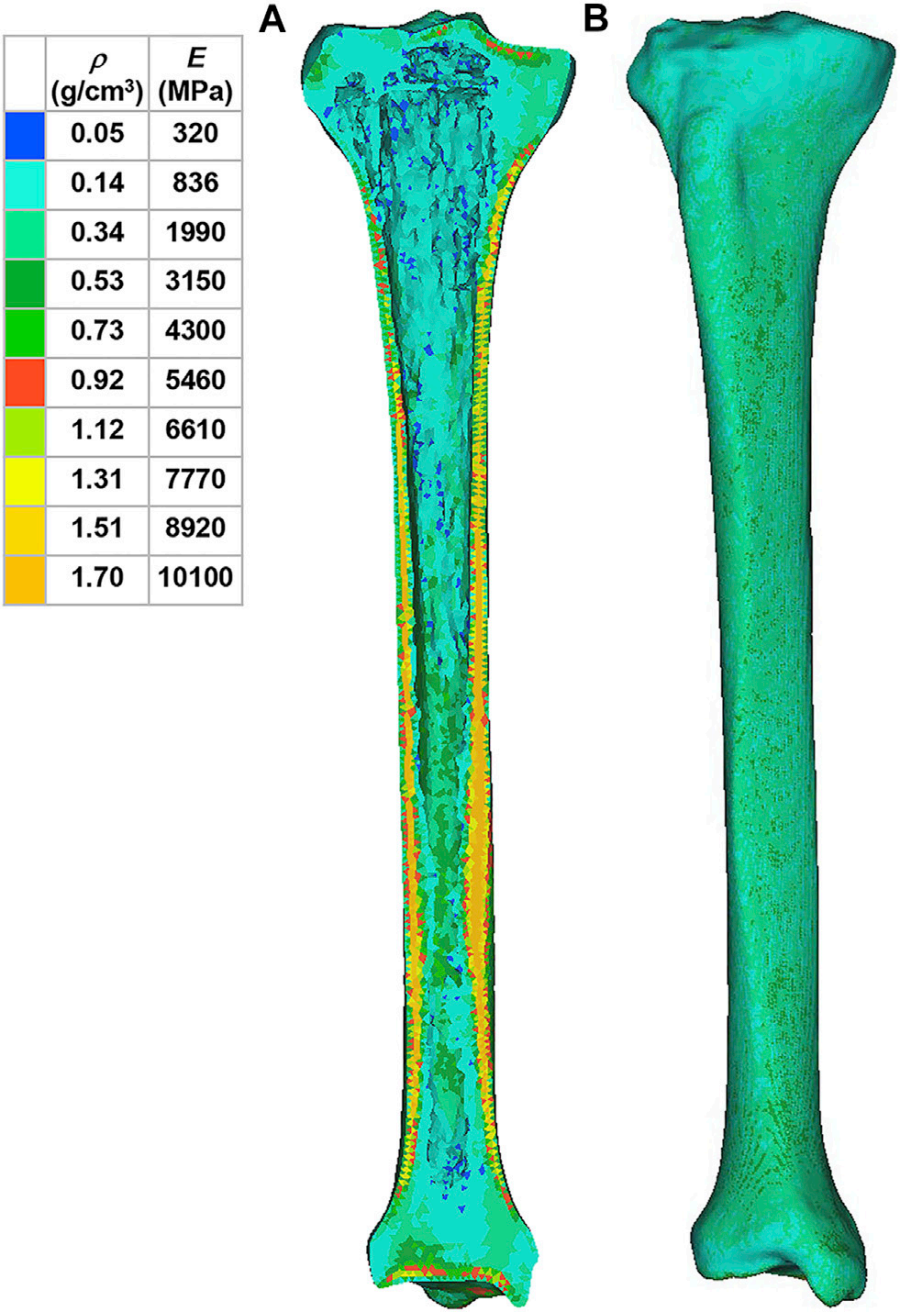
Material properties of the inhomogeneous tibia. **(A)** Internal material properties of the tibia. **(B)** External material properties of the tibia. *ρ*: bone density. *E*: elastic modulus.

Using Mimics software, the 3D tibia model was defined with inhomogeneous material properties according to the gray values of the CT scan. According to previous literature, the material properties of the tibia were determined based on the following formulae (Mo et al., 2019):

$$\rho \left(g/m^{3}\right)=-13.4+\mathrm{1017}\times \mathrm{GV}\left(\mathrm{HU}\right),$$ (1)

$$E\left(\mathrm{Pa}\right)=-388.8+5925\times \rho \left(g/m^{3}\right),$$ (2)

where *ρ* is the bone density, GV is the gray value of the bone in CT data, and *E* is the elastic modulus. The material was assumed as linear elastic in the analysis. According to another previous study, Poisson’s ratio of the bone was set to 0.3 (Thompson et al., 2016). In order to reduce the computational time, the material properties of the tibia were divided into 10 parts with different colors to distinguish (Figure 2). The material properties of the implants and cement were derived from previous literature (Table 1) (Chan et al., 2014).

**TABLE 1:** Material properties of the tibial components.

Prosthesis component	Material	Elastic modulus (MPa)	Poisson’s ratio
Tibial tray and stem	Ti6Al4V	110000	0.34
Tibial insert	UHMWPE	500	0.40
Bone cement	PMMA	2500	0.38
Metal block augmentation	Ti6Al4V	110000	0.34
Optimized part of the block	Ti6Al4V	63000	0.34
Removed part of the block	Ti6Al4V	20000	0.34

### Meshing and Load Setting

All components were imported into Hypermesh v14.0 software (Altair Engineering, Troy, MI, United States) to generate triangular meshes and tetrahedral elements. The element size of the tibia model was set to 1 mm. Owing to the small features in the cement, block, tibial tray, stem, and insert, their element sizes were set to 1, 1, 0.59, 0.48, and 0.36 mm, respectively. A surface-to-surface contact type was set within the different contact surfaces of these components. The tray–cement, block–cement, stem–cement, bone–cement, and tray–insert interfaces were set as freeze contacts, which means enforced zero relative displacements on the contact interface. Sticky contact was set for the remaining interfaces such as tray–stem, tray–block, and block–bone structures. Sticky connection is an enforced stick condition during which the contact interfaces will not enter the sliding phase.

**FIGURE 3:**
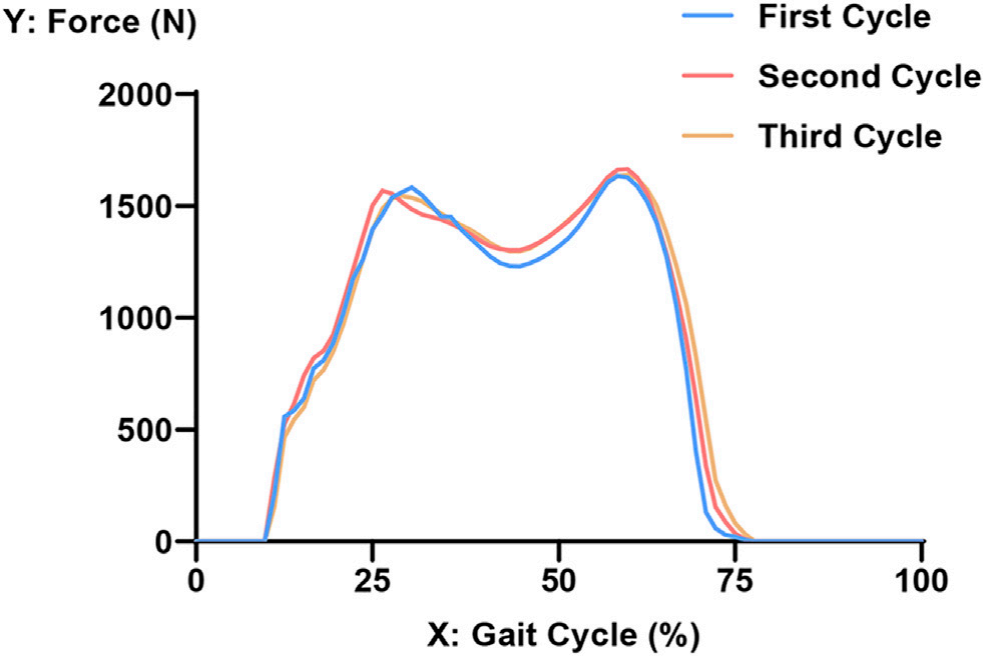
Longitudinal compression force of the right knee during three gait cycles. The x-axis refers to the process of an entire gait cycle. A value of 0 on the Y-axis represents the segment where only the left foot makes contact with the ground. Furthermore, 10% of the gait cycle refers to the initial contact moment of the right heel, and 75% of the gait cycle refers to the right toe-off moment. The peak force is at about 60% of the gait cycle.

**FIGURE 4:**
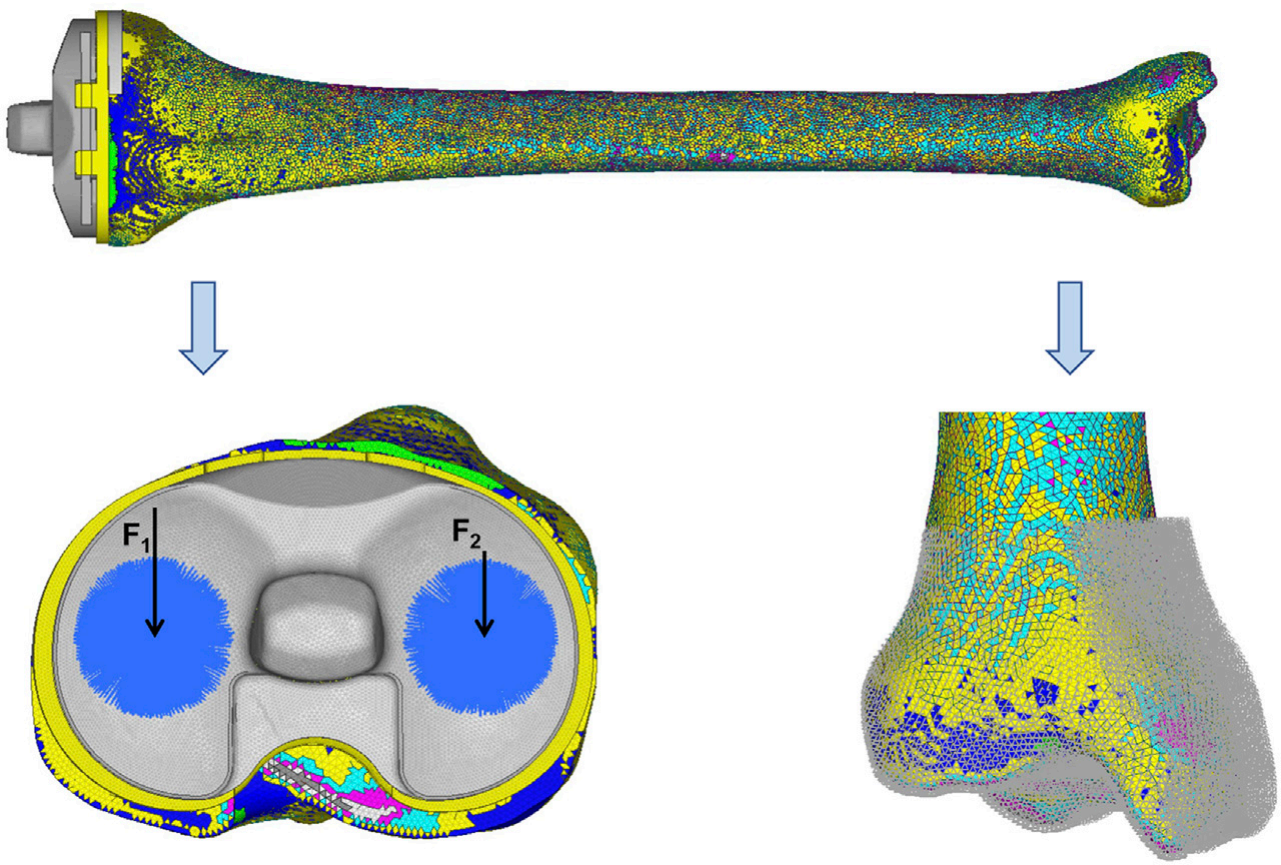
Loads and constrains. F_1_ (987 N) and F_2_ (658 N) were set as the axial forces of the medial and lateral parts on the tibia insert, respectively. The distal tibia was constrained in all directions.

The load was acquired from the gait analysis of the study volunteer preoperatively, using Cortex v5.5.0 software (Motion Analysis, Santa Rosa, CA, United States). This motion capture system consists of six cameras covering 360°, four force plates under the gait path, and 19 markers on the lower limbs of the volunteer. The data were collected and analyzed using Orthotrak v6.1.1 software (Motion Analysis). The longitudinal compression forces of the right knee during the whole gait cycle at three different time points are presented in Figure 3. The average peak force was 1645 N. According to relevant literature, the ratio of the medial and lateral platform load is 60%: 40%. Thus, the axial force of the medial and lateral parts on the tibial insert was set to 987 and 658 N, respectively (Hogel et al., 2013; Lin et al., 2017). “Rigid bar element 3 (rbe3)” function was used in Hypermesh to evenly distributed the force to the medial and lateral parts of the tibial insert, as shown in Figure 4. First, a number of nodes were chosen in a circle, and a main node in the center was automatically created. Then, when the load was added to the main node, it was evenly distributed to the abovementioned nodes. The distal tibia was fixed in all directions.

### Mesh Convergence

Sensitivity analysis of the mesh density of the tibia model was carried out to verify whether the model predictions were affected by mesh refinement (Shriram et al., 2017). Owing to the small features in the other components, the element sizes of cement, block, tibial tray, stem, and insert were set to 1, 1, 0.59, 0.48, and 0.36 mm, respectively. The element size of the tibia was varied to yield four different mesh resolutions, by using very refined mesh as the reference for comparison (Table 2). In the reference case, the element size of the tibia was 0.5 mm. In cases a, b, and c, the element size of tibia was 1, 1.5, and 2 mm, respectively. The peak von Mises stress values of the tibia predicted by cases a–c were compared with that predicted by the reference case, and the cases within ±5% of the reference case were considered as accurate. Case a was found to be optimal, as it required less computational time while maintaining 98% prediction accuracy than the reference case model. The predictions by cases b and c were inaccurate (>5%).

**TABLE 2:** Sensitivity analysis on the mesh density of the tibia.

Case	Element size (mm)	Number of elements	% Change in peak stress
Reference	0.5	3304208	—
Case a	1	689082	2
Case b	1.5	275930	6.8
Case c	2	145369	9.6

### TO of Metal Block Augmentation

The TO was performed in Hypermesh. Minimum compliance TO under the constraint of volume fraction was adopted based on previous literature (Chen and Shih, 2018). The optimization equation is as follows:

Objective function: minimize (U_c_)

*Constraint:* 0 < η_i_ < 1 (i = 1, 2, 3…n)

$$V\leq V_{0}-V*,$$ (3)

$$V=\sum_{i}\eta_{i}V_{i},$$ (4)

$$E_{i}=E\left(\eta_{i}\right),$$ (5)

$$\left\{\sigma_{i}\right\}=\left[E_{i}\right]\left\{\varepsilon_{i}\right\},$$ (6)

where *U*_*c*_ is the compliance, η_***i***_ represents the internal pseudo-densities assigned to each finite element (i) in the optimization equation, V is the computed volume, V_0_ is the original volume, V* represents the amount of volume to be removed, V_i_ is the volume of element i, E_i_ is the elasticity tensor for each element, E represents the elasticity tensor, σ_i_ is the stress vector of element i, and εi represents the strain vector of element. η, as the density index, ranged from 0 to 1. An η value close to 0 indicates that the material is to be removed, and an η value close to 1 indicates that the material is to be retained. The program was set to reduce the volume by up to 50% and iterate 30 times at most.

In Hypermesh, the optimized part was chosen on the pseudo-density of 0.65. On this pseudo-density, the optimized part was relatively regular and beneficial for post-processing. By performing a Boolean operation between the intact model and the optimized part, the removed part was acquired. The TO results were imported to Magics v21.0 software (Materialise, Leuven, Belgium) to design the internal architecture. The shape of the block will cause stress at the bone–implant interface (Brigstocke et al., 2012). In this study, the newly designed block maintained the shape of the original one. The removed and the optimized part were both designed for the body-centered cubic structure with high strength. The removed part was designed for an optimum porosity of 70% and pore size of 600 μm to allow for early and extensive bone ingrowth (Arabnejad et al., 2016a; Arabnejad et al., 2016b). The optimized part was designed for the porosity of 30% and pore size of 600 μm to maintain stiffness and promote proper bone ingrowth (Taniguchi et al., 2016; Chen et al., 2017). Then, the biomechanical changes compared with the original block were analyzed by FEA in Hypermesh. The material properties of the components with porous architecture were calculated based on the relationship between porosity and elastic modulus (Table 1) (Alkhatib et al., 2019).

The FEA results were processed using Hyperview v14.0 software (Altair Engineering). Von Mises stresses of the block and proximal tibia were recorded. The proximal tibia was divided into the medial and lateral parts. The bone defect area was in the medial part. The proximal medial tibia that contacted with the block was divided into three parts: anterior, medial, and posterior. The average stress of all the nodes in the anterior part represents the average stress of the anterior part. The definition was applied to the left two parts. Statistical analyses were performed using SPSS v21.0 software (IBM, Armonk, NY, United States). The comparison between the original and optimized groups was analyzed by using the paired samples *t*-test, with *p* < 0.05 indicating statistically significant differences. Strain energy density (SED) was used as the mechanical stimulus and could be used as an index of stress shielding. Inserting relatively stiff implants into the bone will result in a nonphysiological distribution of load and a decrease in periprosthetic bone strain (Ahmed et al., 2020). Where the same load was first carried by the bone, it is now carried by the prosthetic and the bone. As a consequence, the bone is subjected to reduce stress, hence stress shielded (Huiskes et al., 1992). Moreover, when the stress shielding happens, the strain of the periprosthetic bone will decrease. Thus, high SED indicates low stress shielding (Zhang et al., 2020). Also, the SED has been used as the stimulus to drive bone resorption (Fraldi et al., 2010). In this study, the SED of the proximal tibia was obtained to assess the effectiveness of the optimized block for reducing stress shielding.

## Results

### Topology Optimization Results

**FIGURE 5:**
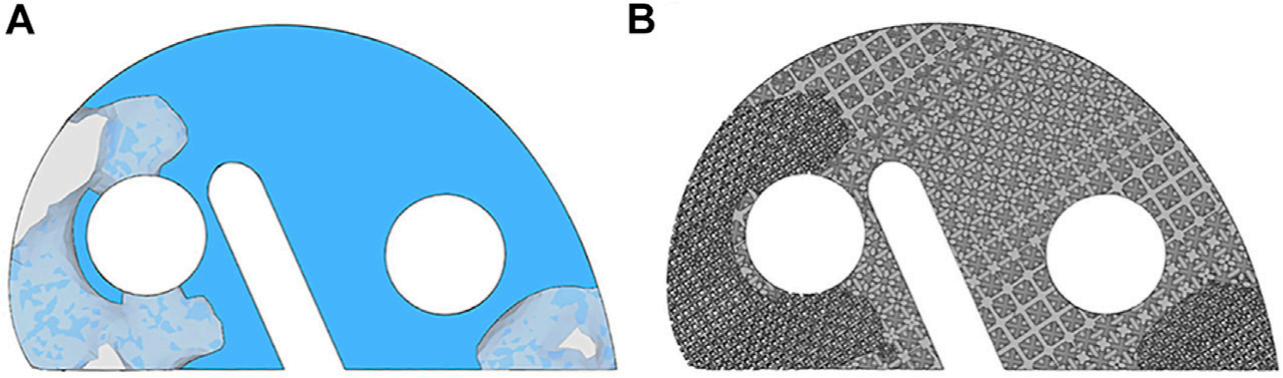
Illustration of the topology optimization results and the newly designed porous metal block augmentation. **(A)** Topology optimization results. The component in the solid and the light shaded blue is the optimized part, and the remaining is the removed part. **(B)** Newly designed porous metal block augmentation.

The TO program iterated 5 times, and the results are shown in Figure 5A. The anterior and posterior parts were mainly removed by the algorithm. About 75% of the original metal block was preserved. Figure 5B shows the newly designed metal block augmentation with porous architecture; the removed part was designed for the porosity of 70%, and the optimized part was designed for the porosity of 30%. This newly designed block achieved 40% weight reduction.

### Von Mises Stress of the Metal Block Augmentation

**FIGURE 6:**
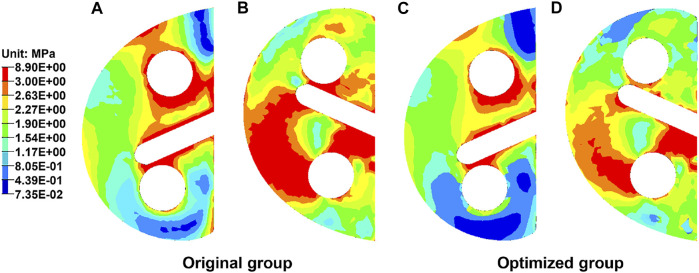
Distribution of the von Mises stress in the metal block augmentation from top and bottom views. **(A)** Original block from the top view. **(B)** Original block from the bottom view. **(C)** Optimized block from the top view. **(D)** Optimized block from the bottom view.

Figure 6 exhibits the von Mises stress of the original and optimized blocks from both top and bottom views. The maximum stress of the original block was 8.90 MPa and that of the optimized one was 7.87 MPa, indicating a decrease of 11.6%. The maximum stress of the optimized block was lower than the yield strength of the lattice structure for 70% porosity (132 MPa) (Wang et al., 2019). The average stress of all the nodes in the original block (2.18 ± 1.05 MPa) was significantly greater than that in the optimized block (1.90 ± 0.88 MPa) (*p* < 0.05). In the top view, the anterior and middle parts were subjected to higher stresses. In the bottom view, the medial and middle parts were subjected to higher stresses. Furthermore, the area of high stress was smaller in the optimized block.

### Von Mises Stress of the Proximal Tibia

**FIGURE 7:**
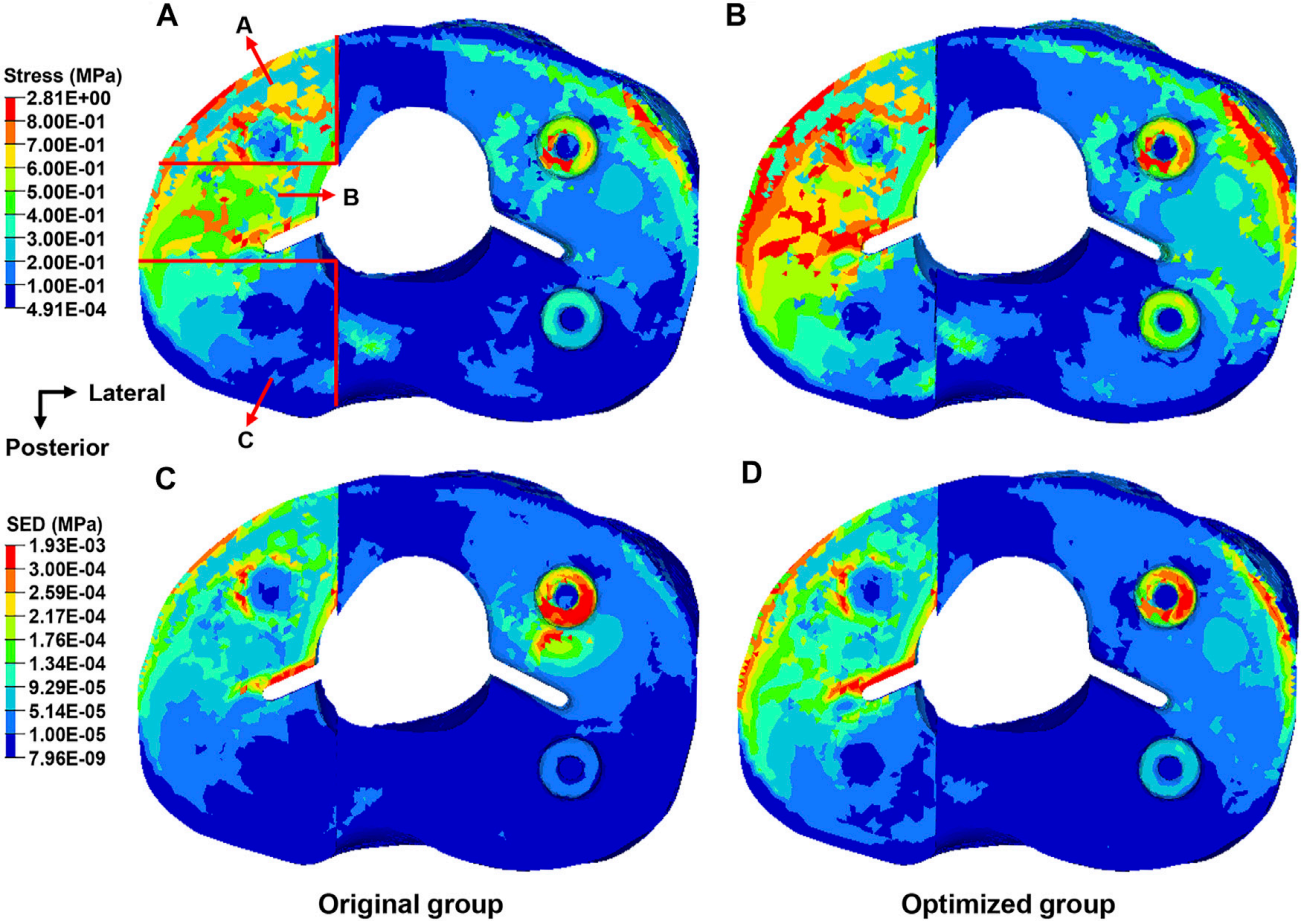
Distribution of the von Mises stress and strain energy density in the proximal tibia from the top view. **(A)** Distribution of the stress in the original group. **(B)** Distribution of the stress in the optimized group. **(C)** Distribution of the strain energy density in the original group. **(D)** Distribution of the strain energy density in the optimized group.

Von Mises stress of the proximal tibia is shown in Figure 7. The maximum stress in the proximal tibia of the original group was 2.37 MPa and that in the optimized group was 2.81 MPa, indicating an increase of 18.6%. The bone defect area was divided into three parts. Table 3 shows the average stress of the anterior, middle, and posterior parts. The stress of each part in the optimized group was significantly greater than that in the original group (*p* < 0.05). The stress concentration area was larger in the optimized group than the original group.

**TABLE 3:** Average stress of the anterior, middle, and posterior parts in the proximal medial tibia (mean ± SD, MPa).

Group	Anterior	Middle	Posterior
Original group	0.42 ± 0.25	0.43 ± 0.23	0.15 ± 0.13
Optimized group	0.44 ± 0.27	0.56 ± 0.30	0.23 ± 0.19

### SED of the Proximal Tibia

The SED in the proximal tibia of the original and the optimized group is shown in Figure 7. The maximum SED in the proximal tibia of the original group was 1.43 kPa and that in the optimized group was 1.93 kPa, corresponding to an increase of 35.0%.

## Discussion

As one of the most common treatments for uncontained bone defects in TKA, metal block augmentation is usually created as a solid structure with high stiffness, which will cause significant alteration in stress distribution and is not suitable for osseointegration (Chung et al., 2016). The most severe problem caused by this alteration is stress shielding. As time goes by, the stress shielding will cause bone resorption and result in aseptic loosening around the prosthesis (Rahimizadeh et al., 2018). Also, the fully solid structure without osseointegration is not beneficial for long-term stability. In this study, a porous metal block augmentation was designed to improve its biomechanical performance based on the TO technique and porous architecture. The biomechanical changes compared with the original block were analyzed by FEA.

An inhomogeneous tibia model was reconstructed to simulate the real tibia in this study. The material properties of the tibia were assigned based on the gray values of the CT scan. The inhomogeneous tibia model adopted in this study could improve the accuracy of the FEA. Ahmet et al. explored the relevance of inhomogeneity in the tibia and reported that the inhomogeneous tibia would cause a substantial difference in the stress values compared with the rigid one (Venäläinen et al., 2016; Ün and Çalık, 2016). On the other hand, choosing an appropriate method to acquire the load is essential for the FEA. In this study, the load data were not directly derived from the previous literature: Gait analysis of the volunteer was conducted to acquire the maximum load on the tibial platform to mimic the extreme situation in the level walking condition. Shu et al. (2018) reported that the maximum medial and lateral contact forces were about 1050 and 550 N, respectively, which appeared at the second force peak. The peak force during the whole gait cycle was 1645 N and appeared at the second force peak in this study, which was close to Shu’s results. Crocombe et al. used FEA to evaluate the biomechanical performance of different reconstructive techniques and showed that the middle region of the proximal lateral tibia in the metal block augmentation group was subjected to higher strains (Completo et al., 2013). The middle region of the proximal lateral tibia is of low stiffness, so relatively low stresses and high strains will be produced in this region. In this study, the stress in the proximal lateral tibia was predominantly located on the cancellous bone, which was similar to the results of Crocombe et al.

Biomechanical changes compared with the original block were analyzed by FEA. Von Mises stress could effectively reflect the biomechanical characteristics, and the stress distributions of the block and proximal tibia were recorded (Thompson et al., 2016). As shown in Figure 6, the maximum stress of the block in the optimized group decreased by 11.6%. The maximum stress of the optimized block (7.87 MPa) was far less than the yield strength of the Ti6Al4V material with 70% porosity (132 MPa) (Wang et al., 2019). Thus, the optimized block will not begin to approach failure levels. Moreover, the red region in the cloud diagram, where the stress was >2.63 MPa, became distinctly smaller in the optimized block (Figure 6). This showed that the stress on the block had been effectively reduced. Previous studies reported the radiolucent line beneath the block as a shortcoming in TKA, which might be due to bone resorption (Guha et al., 2008; Hamai et al., 2015). Stress shielding can cause bone resorption, resulting in the radiolucent line. In this study, the maximum stress of the proximal tibia in the optimized group was 18.6% higher than that in the original group (Figure 7). The stress of the anterior, medial, and posterior parts in the proximal medial tibia from the optimized group was significantly greater than that in the original group (*p* < 0.05). The increased stress will reduce the bone resorption caused by stress shielding, and the occurrence rate of the radiolucent line will decrease. The SED is another index for evaluating stress shielding. The difference in SED between the original and the optimized group could be used to evaluate tibial bone resorption. In this study, the maximum SED of the proximal tibia was 1.43 kPa in the original group and 1.93 kPa in the optimized group, which might indicate the decrease in bone resorption and stress shielding. The abovementioned results indicated the effectiveness of the newly designed porous metal block augmentation in improving its biomechanical performance.

This study has some limitations. First, biomechanical changes compared with the original block were only analyzed by FEA. Although FEA supported the effectiveness in reducing stress shielding, the newly designed block requires further experimental corroboration. The fabrication and biomechanical trials of the newly designed block will be conducted in future studies. Second, only one TKA model was included in this study, which will limit the clinical and design applicability. More samples will be considered in future studies. Third, gait analysis was conducted to derive the knee joint kinetics. However, there is debate of the accuracy of the inverse dynamic techniques utilized to calculate the loads. Moreover, for technical reasons we only used the level walking condition for gait analysis. Other types of movement are important for daily life activities such as squatting and stair climbing. This area needs further studies to increase the accuracy.

## Conclusion

In this study, TO and porous architecture were jointly used to redesign a porous metal block augmentation to improve its biomechanical performance. The newly designed block achieved 40% weight reduction, and the biomechanical changes compared with the original block were analyzed by FEA. The maximum stress in the optimized block decreased by 11.6% when compared with the original one. The maximum stress of the proximal tibia in the optimized group increased by 18.6%, and the stress of the anterior, medial, and posterior parts of the proximal medial tibia in the optimized group was significantly greater than that in the original group (all *p* values < 0.05). The maximum SED of the proximal tibia in the optimized group increased by 35.0%. The newly designed porous block could effectively improve the biomechanical performance between the block and bone. The presented method might provide a reference for the design of customized 3D-printed prostheses.

## Data Availability

The original contributions presented in the study are included in the article/Supplementary Material; further inquiries can be directed to the corresponding authors.
